# The Uses and Limitations of Condensed Milk as an Infant Food

**Published:** 1897-11

**Authors:** 


### THE USES AND LIMITATIONS OF CONDENSED MILK AS AN INFANT FOOD.

In an editorial, the *Archives of Pediatrics* for August, 1897, says:

If condensed milk is an improper food for infants, is it so irreparably bad that it can not be changed or fortified so as to render it a desirable food? We should say that it can not be made a desirable food; it may be made permissible. In many cases it is the only available food, and in some cases the most desirable that can be obtained. While granting this, we do not in the slightest degree advise its use when a better food can be obtained. It is certainly a fact that the practitioner is sometimes obliged to use it; this occasionally occurs through obstinate persistency on the part of parents, but more commonly among the extreme poor, who can not afford a more expensive food.

As the chief objections to condensed milk as an infant food are its deficiency in fat and proteids, two changes must be made to render it suitable for use: fat and proteid must be added. As the absence of fat is the greater defect of the two, it must receive chief attention. This deficiency may be corrected by the addition of cream—an impossibility among the very poor. If cream is not available we may resort to cod-liver oil; it is an excellent substitute and should be regarded as a food rather than a medicine, and must be given continuously, though the daily amount need not be large. The device of using a meat broth as suggested by Dr. Kerley for securing the proteid is an excellent one. As an occasional substitute for the broth, egg albumen may be utilized to supply the necessary nitrogen; the white of an egg may be thoroughly beaten up with the water with which the condensed milk is diluted. The chief objection to this plan is the difficulty of determining the proper proportions to be employed. By thus modifying condensed milk a child may frequently be carried with fair success to the ninth month. His chances, however, of reaching that age without rickets will be far better with fresh cow's milk. One advantage, it must be acknowledged, in the use of the condensed milk is the fact that the child is less liable to be fed with an overstrong mixture than when fresh milk is used. One of the most frequent and serious errors is overfeeding. The fact that children do no worse on these excessively weak condensed milk mixtures is but one of many proofs that they commonly receive more food than they require. If the doctor who is wedded to the exclusive use of condensed milk would not make his fresh milk mixtures four to six times as strong as his condensed milk mixture, he would be much better satisfied with fresh milk. In deciding upon the value of a given food the physician should not fix his attention upon the present so closely as to entirely forget the future. He should consider the remote, as well as the immediate, effects of the diet. His office is not alone to tide over a few months and keep a baby quiet at any hazard, but to lay the foundation for strong and vigorous childhood. He will fail to accomplish this if he prescribes a food lacking in its essential elements, though the child may for a few months seem to digest it more readily.

